# Left Atrial Appendage Exclusion via Right Minithoracotomy Using an Epicardial Clip Device During Minimally Invasive Mitral Valve Surgery

**DOI:** 10.3390/medicina62071417

**Published:** 2026-07-22

**Authors:** Razan Salem, Pawel Nawrocki, Andreas Däuwel, Feras Kabbesh, Hamid Naraghi Taghi Of, Mohamed Zeriouh, Bujar Maxhera, Mahmoud Diab, Diyar Saeed

**Affiliations:** 1 Department of Cardiovascular Surgery, Heart Center Niederrhein, Helios Klinikum Krefeld, 47805 Krefeld, Germany; razan.salem@helios-gesundheit.de (R.S.);; 2 Department of Humanmedizin, Health and Medical University Duesseldorf/Krefeld, 40221 Duesseldorf, Germany; 3 Department of Humanmedizin, University Witten/Herdecke, 58448 Witten, Germany; 4 Department of Anesthesiology, Helios Klinikum Krefeld, 47805 Krefeld, Germany

**Keywords:** left atrial appendage, LAA exclusion, AtriClip Pro, minimally invasive mitral valve surgery, right minithoracotomy, atrial fibrillation, stroke prevention, epicardial clip

## Abstract

*Background and Objectives*: Left atrial appendage (LAA) closure is a Class I recommendation in patients with atrial fibrillation to reduce the risk of cardioembolic stroke. Achieving reliable and complete LAA exclusion during minimally invasive mitral valve surgery via right minithoracotomy remains technically challenging. We report here to our knowledge the largest series of a novel technique for LAA exclusion using an epicardial clip device applied via right minithoracotomy during minimally invasive mitral valve surgery. *Materials and Methods*: Between June 2023 and May 2026, 40 patients with atrial fibrillation underwent minimally invasive mitral valve surgery via right minithoracotomy with concomitant LAA exclusion. Cardiopulmonary bypass was established via percutaneous femoral cannulation. Following completion of the intracardiac procedure and prior to aortic cross-clamp removal, a suture was placed around the LAA base via the transverse sinus and used to guide clip deployment under direct vision. Successful closure was confirmed by intraoperative transesophageal echocardiography. *Results*: Mean patient age was 66.6 ± 8.0 years; 21 patients (53%) were female. Mitral valve repair was performed in 36 patients (90%) and replacement in 4 (10%). Concomitant cryoablation for AF was performed in 31 patients (78%). Successful LAA clip deployment was achieved in all 40 patients (100%). The 35 mm clip was used in 36 patients (90%), the 40 mm clip in 3 patients (8%), and the 45 mm clip in 1 patient (2%). Mean total operative time was 183 ± 58 min; mean CPB time was 134 ± 42 min; mean aortic cross-clamp time was 70 ± 27 min. In-hospital mortality was 0%. One patient (3%) required re-thoracotomy for bleeding, one developed a postoperative stroke, and two required ECMO support. Median hospital stay was 9 days. At discharge, 18 patients (45%) were in sinus rhythm; among the 31 who underwent concomitant cryoablation, 16 (52%) were discharged in sinus rhythm. *Conclusions*: Minimally invasive LAA exclusion is feasible and safe when performed via right minithoracotomy during minimally invasive mitral valve surgery. The technique achieves high rates of successful deployment and avoids the need for additional incisions or access sites. This approach represents a valuable addition to the armamentarium of concomitant stroke prevention strategies in patients with AF undergoing minimally invasive valvular surgery.

## 1. Introduction

Atrial fibrillation (AF) is the most common sustained cardiac arrhythmia, affecting an estimated 37.5 million individuals worldwide, and carries a fivefold increased risk of ischemic stroke [1]. The left atrial appendage (LAA) is the predominant source of cardioembolism in AF, accounting for more than 90% of thrombi in patients with non-valvular AF and approximately 57% in those with valvular disease [2]. Mechanical exclusion of the LAA at the time of cardiac surgery has emerged as an important strategy for long-term stroke risk reduction, and current STS/AHA as well as ESC/EACTS guidelines assign a Class I recommendation to surgical LAA closure in patients with AF undergoing open cardiac procedures [3,4].

Suture-based LAA closure, historically the most common intraoperative technique, has been shown to be unreliable, with incomplete closure rates as high as 36–60%, predominantly due to the formation of a residual LAA stump [5,6]. These incomplete closures may paradoxically increase stroke risk by creating a low-flow nidus for thrombus formation. External epicardial clip devices—in particular the AtriClip system (AtriCure, Mason, OH, USA)—have demonstrated superior and durable closure rates exceeding 95% in both open and minimally invasive settings, and have become the preferred technique at many high-volume centers [7,8].

Minimally invasive mitral valve surgery via right minithoracotomy is now well established, offering equivalent repair durability to sternotomy while reducing blood transfusion requirements, sternal morbidity, and recovery time [9,10]. The combination of mitral valve surgery with concomitant LAA closure and ablation for AF represents a comprehensive, “one-stop” approach to managing the full spectrum of pathology in these patients. However, the technical execution of LAA Clip deployment through the limited operative field of a right minithoracotomy has not been widely described, and concerns exist regarding feasibility, visualization, and complete closure confirmation in this setting.

The AtriClip Pro device is a second-generation epicardial clip with a flexible, low-profile design and an integral suture channel that facilitates guided deployment [11]. Literature review has shown that only small series are available on minimally invasive LAA exclusion [12]. Therefore, the aim of this study is to report to our knowledge the largest published series on LAA exclusion through the transverse sinus via right minithoracotomy during mitral valve surgery.

## 2. Materials and Methods

### 2.1. Study Design and Patient Population

This is a single-center, retrospective cohort study of all consecutive patients who underwent minimally invasive mitral valve surgery via right minithoracotomy with concomitant LAA exclusion using the AtriClip Pro device at our institution between June 2023 and May 2026. Indication for LAA closure included preoperative AF, paroxysmal or persistent. The study was approved by the local institutional review board (Reference number: 2026058; approval date: 4 May 2026); individual informed consent was waived given the retrospective design. All data were de-identified prior to analysis.

### 2.2. Surgical Technique

All patients underwent right lateral minithoracotomy through the fourth or fifth intercostal space. Cardiopulmonary bypass (CPB) was established via percutaneous ultrasound-guided femoral artery and vein cannulation [13]. Standard endoscopic visualization was used throughout. The mitral valve was accessed via a left atriotomy parallel to the interatrial groove. Following completion of the mitral valve procedure—and while the heart remained arrested with the aortic cross-clamp in place—attention was directed to the LAA.

The LAA was identified through the transverse sinus. Suctioning on the aortic root vent facilitates manipulation of the aorta. A 4-0 polypropylene suture is passed around the tip of the LAA via the transverse sinus, looped through the suture channel of the AtriClip Pro device, and used to guide and seat the clip at the LAA ostium under direct vision. The clip was then deployed, and the guiding suture was removed (Video S1). Complete LAA closure and absence of residual flow were confirmed by intraoperative transesophageal echocardiography (TOE) after the aortic cross-clamp was released and cardiac activity resumed. Concomitant procedures were performed according to the standard institutional protocols. Left atrial cryoablation using a cryoprobe was performed in patients with preoperative AF. TV repair (annuloplasty) was performed in patients with significant secondary TR, mostly on the beating heart.

### 2.3. AtriClip Pro Device

**Figure 1:**
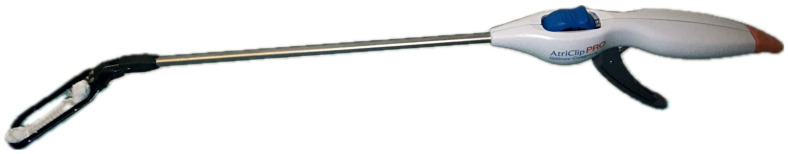
AtriClip Pro by AtriCure.

The AtriClip Pro (AtriCure, Mason, OH, USA) is a second-generation self-closing epicardial clip constructed from a nitinol frame with two parallel rows of polyester mesh-covered arms (Figure 1). The device features an integral suture-guidance channel that allows controlled, guided positioning at the LAA base without requiring direct manual manipulation of the appendage. It is available in 35 mm, 40 mm, and 45 mm sizes, selected based on TOE measurement of the LAA ostium diameter prior to deployment.

### 2.4. Data Collection and Definitions

Preoperative variables recorded included age, sex, BMI, cardiac rhythm, left ventricular ejection fraction (graded as good [≥55%], mildly-to-moderately reduced [30–54%], or severely reduced [<30%]), and preoperative renal function (creatinine, estimated glomerular filtration rate [eGFR]). Operative variables included type of mitral valve procedure, concomitant procedures, clip size, clip deployment success, total operative time, CPB time, and aortic cross-clamp time. Postoperative outcomes included in-hospital mortality, stroke, re-thoracotomy for bleeding, ECMO requirement, new permanent pacemaker implantation, vascular complications, hospital length of stay, discharge rhythm, and pericardial effusion on discharge echocardiography. Long-term follow-up was conducted via telephone contact with patients.

Successful LAA closure was defined as complete occlusion of the LAA ostium on TOE with no residual flow detectable by color Doppler imaging and a residual stump length of less than 1 cm. Failed deployment was defined as inability to position and close the clip safely due to anatomical or technical obstacles.

### 2.5. Statistical Analysis

Continuous variables are reported as mean ± standard deviation (SD) or median (range) according to distribution. Categorical variables are reported as absolute numbers and percentages. Long-term follow-up was obtained through structured telephone contact with patients or their next of kin. Time-to-event was calculated from the date of surgery to the date of the outcome event or last follow-up contact. Kaplan–Meier analysis was performed to estimate freedom from all-cause mortality and freedom from stroke. Patients without a recorded follow-up call at the time of analysis were censored at the date of hospital discharge. Perioperative events were assigned to the day of occurrence during the index admission. Kaplan–Meier curves were truncated at 36 months, at which point fewer than 10 patients remained at risk. Confidence intervals were calculated using the log-log transformation method. No formal inferential statistical testing was performed given the descriptive scope of the study. All statistical analyses were performed using Python v3.11 with pandas and NumPy libraries.

## 3. Results

### 3.1. Patient Characteristics

Forty patients underwent minimally invasive mitral valve surgery with concomitant AtriClip Pro LAA exclusion between June 2023 and May 2026. Baseline characteristics are summarized in Table 1. The mean age was 66.6 ± 8.0 years (range: 48–81), and 21 patients (53%) were female. Preoperative AF was present in 37 patients (92.5%). Preoperative left ventricular function was preserved in 29 patients (73%), mildly-to-moderately reduced in 10 (25%), and severely reduced in 1 (3%).

While the primary indication for concomitant LAA exclusion in this series was preoperative atrial fibrillation, present in 37 of 40 patients (92.5%), three patients received AtriClip Pro deployment in the absence of persistent AF at the time of surgery. Two of these patients had pre-existing pacemakers and presented with atrial fibrillation at admission, with device-documented paroxysmal AF episodes confirming the cardioembolic substrate. The third patient presented in sinus rhythm following a single documented AF episode but carried a high CHA_2_DS_2_-VASc score, and the decision for prophylactic LAA exclusion was made following individualized multidisciplinary assessment. This approach reflects the evolving concept that LAA exclusion should not be restricted to patients with overt AF at the time of surgery, but may be extended to those with paroxysmal or device-detected AF, as well as high-risk patients with a documented AF history, in whom the incremental risk of the procedure during concomitant cardiac surgery is negligible.

### 3.2. Intraoperative Results

Mitral valve repair was performed in 36 patients (90%); mitral valve replacement was required in 4 patients (10%). Concomitant cryoablation for AF was performed in 31 patients (78%). Isolated mitral valve surgery combined with ablation and AtriClip placement was performed in 23 patients (58%). Concomitant Tricuspid valve repair was performed in 14 patients (35%).

AtriClip Pro deployment was successfully achieved in all 40 patients (100%). There were no cases in which LAA exclusion was intended but aborted intraoperatively—the reported 100% procedural success therefore reflects not only successful device deployment, but the absence of any intraoperative failure or conversion to an alternative closure strategy. The 35 mm clip was used in 36 patients (90%), the 40 mm clip in 3 patients (8%), and the 45 mm clip in 1 patient (2%). Clip size was guided by preoperative and intraoperative TEE measurement of the LAA ostium.

One patient (3%) required intraoperative conversion from minithoracotomy to median sternotomy due to an iatrogenic aorto-ventricular dissection encountered during mitral valve re-repair; this patient subsequently underwent successful mitral valve replacement via sternotomy. Mean total operative time was 183 ± 58 min (range: 106–366). Mean CPB time was 134 ± 42 min (range: 78–277) and mean aortic cross-clamp time was 70 ± 27 min (range: 13–158). Operative details are summarized in Table 2.

### 3.3. Postoperative Outcomes

In-hospital mortality was 0%. All postoperative outcomes are detailed in Table 3. Median hospital stay was 9 days (mean: 12.7 ± 11.6; range: 6–67).

Re-thoracotomy for postoperative bleeding was required in one patient (3%). Two patients (5%) required ECMO support: one peri-operatively for low-output cardiac failure in the context of severely reduced preoperative left ventricular function, and one required both preoperative and postoperative ECMO implantation for severe preoperative cardiac decompensation. One patient (3%) experienced a postoperative ischemic stroke. One patient (3%) developed complete atrioventricular block requiring new permanent pacemaker implantation, and one patient (2.5%) sustained access-site vascular complication requiring femoral artery repair and hematoma evacuation.

At discharge, 18 patients (45%) were in sinus rhythm, 15 patients (38%) had persistent AF, and 4 patients (10%) had a pacemaker-dependent rhythm. Among the 31 patients who underwent concomitant cryoablation, 16 (52%) were discharged in sinus rhythm.

### 3.4. Follow-Up and Survival

**Figure 2:**
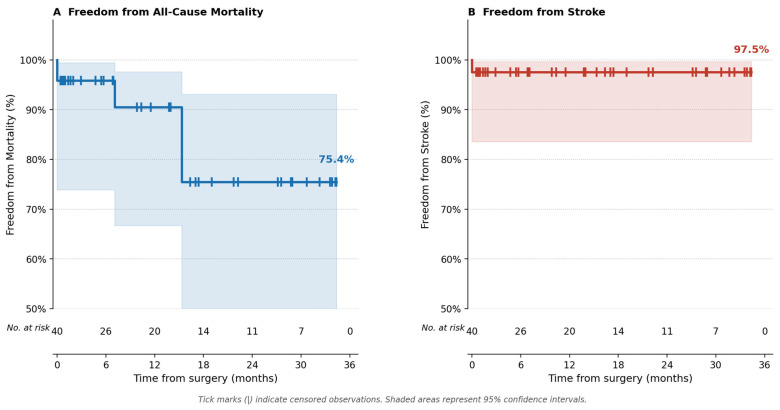
Kaplan–Meier estimates of freedom from all-cause mortality (**A**) and freedom from stroke (**B**) following concomitant left atrial appendage exclusion with the AtriClip Pro device. Tick marks indicate censored observations. Shaded areas represent 95% confidence intervals.

Long-term follow-up was obtained through structured telephone contact and was available for 34 of 40 patients (85%), with the remaining 6 patients—all operated within the final six weeks of the study period—censored at the date of hospital discharge. Median follow-up was 12.6 months (range: 0.5–34.4 months; total: 46.9 patient-years). There was no in-hospital mortality. Kaplan–Meier analysis demonstrated freedom from all-cause mortality of 95.8% (95% CI 73.9–99.4%) at 6 months and 90.5% (95% CI 66.7–97.6%) at 12 months, with all three deaths occurring after discharge (Figure 2A). Freedom from stroke was 97.5% (95% CI 83.5–99.6%) throughout the entire follow-up period, with a single in-hospital stroke event recorded in the early postoperative period (Figure 2B).

## 4. Discussion

In this single-center series of 40 consecutive patients, we demonstrate that LAA exclusion through the transverse sinus using the AtriClip Pro epicardial clip is feasible and safe when performed via right minithoracotomy during minimally invasive mitral valve surgery. The principal findings are: (1) a high successful deployment rate of 100%; (2) no in-hospital mortality was reported; (3) a low overall major complication rate; and (4) a sinus rhythm conversion rate of 52% at discharge among patients who underwent concomitant cryoablation.

The rationale for LAA closure in patients with AF undergoing cardiac surgery is well supported. The 2023 ESC guidelines and the 2023 ACC/AHA guidelines both assign a Class I recommendation to surgical LAA closure at the time of open cardiac procedures in patients with AF, based on growing evidence that mechanical exclusion reduces long-term stroke risk [3]. The landmark LAAOS III randomized trial demonstrated a 33% relative reduction in stroke or systemic embolism with surgical LAA closure in patients with AF undergoing cardiac surgery, providing the highest level of evidence for this practice [14]. Given these data, there is an increasing imperative to offer complete LAA exclusion as a routine component of surgical care for AF patients, including those undergoing minimally invasive procedures.

The present study adds to a growing body of evidence supporting epicardial LAA exclusion as an effective stroke prevention strategy during concomitant cardiac surgery. A recent dual-center study by La Fazia et al. [15] examining TEE-guided endovascular LAAC with the Watchman FLX device highlighted residual peridevice leak (PDL) as a persistent clinical concern, occurring in 32.6% of patients with standard device compression—a rate that was only reduced to 8.2% with deliberate device overcompression (>30%), which itself carries procedural implications. In contrast, the epicardial AtriClip Pro device operates through a fundamentally different mechanism: external mechanical exclusion of the LAA eliminates the anatomical substrate for peridevice flow, and complete closure was confirmed intraoperatively by TEE in all 40 patients in our series. This distinction is clinically relevant, as residual LAA flow—regardless of its magnitude—has been associated with thrombus formation and persistent embolic risk in the endovascular literature. The 97.5% freedom from stroke observed throughout our follow-up period is consistent with this principle and supports the hypothesis that complete, leak-free LAA exclusion at the time of surgery may confer durable stroke protection. Although direct comparison between epicardial and endovascular LAAC techniques is limited by differences in patient population, indication, and follow-up methodology, our findings complement those of Montella et al. in underscoring complete LAA occlusion—by whatever means confirmed—as the primary determinant of clinical efficacy.

The technical challenge unique to our approach is the deployment of the AtriClip via the constrained operative field of a right minithoracotomy. In standard open surgery, the LAA is directly accessible from the anterior pericardium and can be visualized and manipulated without difficulty. In minimally invasive surgery via the right side, however, the LAA is anatomically distant from the primary operative field, is not directly visible through the atriotomy, and must be approached through the transverse sinus. Our technique—in which a guiding suture is placed around the LAA base via the transverse sinus and passed through the clip’s integral suture channel—overcomes these geometrical constraints by providing controlled, tension-guided clip positioning without requiring direct instrument contact with the LAA body. This approach is conceptually analogous to techniques described by other groups using video-assisted or robotic platforms [16,17]. In comparison, the novelty of this technique lies in overcoming a fundamental anatomical challenge inherent to right minithoracotomy: the LAA lies posteriorly displaced and out of direct reach from the right-sided operative field, making epicardial clip deployment—straightforward via median sternotomy—technically demanding in this setting. Transverse sinus access combined with a suture-guided delivery maneuver, as we describe in this series, resolves this constraint, enabling reliable and reproducible AtriClip Pro deployment as a routine concomitant step during minimally invasive mitral valve surgery. While epicardial LAA exclusion has been described in robotic-assisted procedures—a highly specialized approach confined to a small number of expert centers—and in thoracoscopic standalone LAA closure performed via left-sided access with direct LAA visualization, neither translates to the right minithoracotomy platform that represents the global standard for minimally invasive mitral surgery.

The 100% successful deployment rate achieved in our series is consistent with or exceeds published results from open surgical cohorts. A meta-analysis of surgical LAA closure methods demonstrated complete occlusion in 95.7% of epicardial clip cases versus 43–60% for suture ligation [6]. In patients with prior cardiac surgery, pericarditis, or radiation therapy, careful pre-procedural planning is essential, as pericardial adhesions may pose a risk to safe clip placement.

The concomitant cryoablation rate of 78% in our cohort reflects a comprehensive, guideline-concordant approach to rhythm management in AF patients undergoing valvular surgery. Current STS guidelines upgraded the recommendation for concomitant ablation in patients with paroxysmal or persistent AF to a Class I indication in the context of cardiac surgery, while the ESC/EACTS recommendation strength for concomitant surgical ablation is generally considered slightly less prescriptive than the STS 2023 Class I upgrade, with notable variation in indication specificity compared to American guidelines [4,18]. In our series, 52% of ablated patients were discharged in sinus rhythm—a rate consistent with published short-term results from cryoablation series, recognizing that rhythm stability at discharge does not necessarily reflect long-term success and that AF recurrence is common in the early postoperative period [19].

The overall complication profile of our series is in keeping with expected rates for minimally invasive mitral valve surgery in a mixed population that includes complex concomitant procedures. The single vascular access complication (femoral artery thrombosis) is an established risk of percutaneous femoral cannulation, occurring in approximately 1–3% of minimally invasive cardiac surgery series [20]. The one postoperative stroke (2.5%) occurred in a patient with an undiagnosed lung tumor.

## 5. Conclusions

LAA exclusion during minimal invasive cardiac surgery though transverse sinus is feasible, safe, and effective when performed. The technique achieves a high deployment success rate without requiring additional access, incisions, or a change in operative position. The approach integrates naturally into the minimally invasive surgical workflow and can be combined with cryoablation and other concomitant procedures in the same setting. Given the strong guideline recommendations for LAA closure in AF patients undergoing cardiac surgery, this technique provides a reproducible and reliable method for delivering complete stroke prophylaxis as part of a comprehensive minimally invasive valvular intervention.

### Limitations

This study has several limitations inherent to its design. It is a single-center retrospective series with a relatively small sample size and no control group, precluding direct comparison with suture-based LAA closure or other techniques and limiting generalizability across institutions and surgical teams. The heterogeneity of concomitant procedures—ranging from isolated mitral valve repair to complex multi-valve surgery with ablation—introduces variability in operative complexity that may influence outcomes. Regarding follow-up imaging, all patients underwent routine transthoracic echocardiography, which is not suitable for LAA visualization and cannot assess residual flow or closure integrity. Definitive imaging assessment would require transesophageal echocardiography or CT angiography; however, the requirement for sedation and radiation exposure, respectively, is difficult to justify in asymptomatic patients outside of a clinical indication. Intraoperative TEE-confirmed complete closure therefore represents the primary imaging endpoint of this study, and TEE-based assessment of closure adequacy, while standard practice, carries inherent inter-observer variability. Larger prospective multicenter studies with standardized echocardiographic core-laboratory assessment, longer follow-up, and comparative arms are required to definitively establish the efficacy and safety of this approach.

## Figures and Tables

**Table 1:** Baseline patient characteristics.

Characteristic	Value (N = 40)
Age, years, mean ± SD (range)	66.6 ± 8.0 (48–81)
Male sex, n (%)	21 (53)
BMI, kg/m^2^, mean ± SD	26.4 ± 4.1
Height, m, mean ± SD	1.74 ± 0.09
Weight, kg, mean ± SD	80.6 ± 16.3
Preoperative rhythm, n (%)	
Sinus rhythm	1 (2.5)
Atrial fibrillation	37 (92.5)
Pacemaker rhythm	2 (4)
Preoperative LV function, n (%)	
Preserved (EF ≥ 55%)	29 (73)
Mildly–moderately reduced	10 (25)
Severely reduced	1 (3)
Preoperative GFR, mL/min/1.73 m^2^, mean ± SD	71.0 ± 17.8

BMI = body mass index; GFR = glomerular filtration rate; LV = left ventricular.

**Table 2:** Operative details.

Variable	Value (N = 40)
Mitral valve procedure, n (%)	
MV repair (annuloplasty ± neochordae)	36 (90)
MV replacement	4 (10)
Concomitant TV repair, n (%)	14 (35)
Concomitant cryoablation, n (%)	23 (74)
Concomitant PFO closure, n (%)	5 (12.5)
AtriClip Pro size, n (%)	
35 mm	36 (90)
40 mm	3 (8)
45 mm	1 (2)
Successful LAA clip deployment, n (%)	40 (100)
Total operative time, min, mean ± SD (range)	183 ± 58 (106–366)
CPB time, min, mean ± SD (range)	134 ± 42 (78–277)
Aortic cross-clamp time, min, mean ± SD (range)	70 ± 27 (13–158)
Conversion to sternotomy, n (%)	1 (3)

MV = mitral valve; TV = tricuspid valve; LAA = left atrial appendage; CPB = cardiopulmonary bypass; PFO = patent foramen ovale.

**Table 3:** Postoperative outcomes.

Outcome	Value (N = 40)
In-hospital mortality, n (%)	0 (0)
Postoperative stroke, n (%)	1 (3)
Re-thoracotomy for bleeding, n (%)	1 (3)
ECMO support, n (%)	2 (5)
New permanent pacemaker implantation, n (%)	1 (3)
Femoral vascular complication, n (%)	2 (5)
Hospital stay, days, mean ± SD (median)	12.7 ± 11.6 (9)
Discharge rhythm, n (%)	
Sinus rhythm	18 (45)
Atrial fibrillation	15 (38)
Pacemaker-dependent rhythm	4 (10)
SR at discharge among ablation patients (n = 31), n (%)	16 (52)

ECMO = extracorporeal membrane oxygenation; SR = sinus rhythm.

## Data Availability

The data presented in this study are available on request from the corresponding author.

## Supplementary Materials

The following supporting information can be downloaded at: https://zenodo.org/doi/10.5281/zenodo.21471269 (accessed on 13 July 2026), Video S1: Minimally invasive LAA-Closure via Right Mini-thoracotomy using an epicardial clip device (AtriClip Pro) through the transvers sinus.
